# Update on novel acne treatments: a narrative review focused on microbiome modulation and non-pharmacological approaches^☆^

**DOI:** 10.1016/j.abd.2025.501249

**Published:** 2026-01-15

**Authors:** Valentina Burckhardt-Bravo, Rodrigo Funes-Ferrada, Fernando Valenzuela

**Affiliations:** a Department of Dermatology, Faculty of Medicine, Universidad de Los Andes, Santiago, Las Condes, Chile; b Department of Dermatology, Faculty of Medicine, Universidad de Chile, Santiago, Independencia, Chile

**Keywords:** Acne vulgaris, Photochemotherapy, Skin microbiome, Therapeutics

## Abstract

Acne vulgaris is a chronic inflammatory condition with multifactorial pathogenesis. Despite the availability of numerous treatment options, there remains a need for safe, well-tolerated, and microbiome-preserving therapies. This narrative review explores recent advances in non-pharmacological acne treatments, focusing on various microbiome modulation strategies. It highlights emerging therapeutic modalities and their potential impact on clinical practice. Key findings from recent studies are summarized, providing insights for future research and practical applications in dermatology.

## Introduction

Acne vulgaris is a chronic inflammatory skin condition with multifactorial pathogenesis, involving abnormal keratinization, sebum overproduction, inflammation of the pilosebaceous unit, microbial colonization by *Cutibacterium acnes*, and dietary influences.^1^, ^2^ Although numerous effective treatments are currently available, such as retinoids, antibiotics, and hormonal agents, they are often limited by adverse effects, antimicrobial resistance, and disruption of the skin microbiome. These limitations have intensified the search for alternative therapies that can restore microbial balance, modulate host-microbe interactions, and enhance safety and tolerability.^3^

The cutaneous microbiome plays a fundamental role in maintaining skin barrier integrity and regulating local immune responses. Its dysregulation can exacerbate inflammation, impair immune tolerance, and promote the overgrowth of pathogenic microorganisms, mechanisms that are increasingly associated with persistent or treatment-resistant acne.^4^

Consequently, microbiome-targeted approaches have emerged as a promising and innovative field in acne treatment. Advances in understanding the microbiome’s role in cutaneous immunity and inflammation have opened new avenues for developing safer, more personalized therapies that address dysbiosis while preserving microbial diversity.^5^

This narrative review provides an overview of emerging non-pharmacological strategies, including topical probiotics, postbiotics, live biotherapeutic products, biotechnological phytocomplexes, bacteriophages, vaccines, gut-skin-axis interventions, and energy-based modalities such as photodynamic therapy. These strategies aim to achieve clinical efficacy while preserving or restoring microbiome balance, representing a paradigm shift in the contemporary management of acne vulgaris.

## Methods

A comprehensive literature review was conducted to identify recent studies on non-pharmacological treatments for acne, focusing on microbiome modulation strategies. The review included articles published between 2022 and 2024, excluding those focused solely on scar treatments, while considering studies addressing all severities of inflammatory acne vulgaris. The search strategy prioritized clinical trials, Randomized Controlled Trials (RCTs), meta-analyses, and systematic reviews. To ensure relevance, filters were applied to include only studies published between 2022 and 2024. Searches were conducted on December 29, 2024, across multiple databases, including PubMed, Google Scholar, MEDLINE, the Cochrane Central Register of Controlled Trials (CENTRAL), and ClinicalTrials.gov. Additionally, a snowballing technique was employed to identify relevant studies cited in the reviewed literature. This review is based exclusively on previously published research and excludes any original studies conducted by the authors. Table 1^6^, ^7^, ^8^, ^9^, ^10^, ^11^, ^12^, ^13^, ^14^, ^15^, ^16^, ^17^, ^18^, ^19^, ^20^, ^21^, ^22^, ^23^, ^24^, ^25^, ^26^, ^27^, ^28^ summarizes the key studies included in this narrative review.

**Table 1:** Comparative overview of key studies included in this narrative review.^6^, ^7^, ^8^, ^9^, ^10^, ^11^, ^12^, ^13^, ^14^, ^15^, ^16^, ^17^, ^18^, ^19^, ^20^, ^21^, ^22^, ^23^, ^24^, ^25^, ^26^, ^27^, ^28^

Authors / Year	Therapy	Study Design	Patients	Objective	Intervention	Key Findings	Safety	Conclusion
Lebeer et al. / 2022^6^	Topical Probiotics	Double-blind, placebo-controlled clinical trial.	79 patients mild-to-moderate acne.	To evaluate the efficacy and safety of a cream with lactobacilli in modulating the skin microbiome and improving acne.	Topical cream containing *L. rhamnosus* GG, *L. plantarum* WCFS1, *L. pentosus* KCA applied twice daily for 8-weeks.	Reduced *C. acnes* and *Staphylococcus spp.* colonization; increased *Lactobacillus spp.* abundance. Clinically, inflammatory lesions decreased and skin hydration improved.	Well tolerated, mild erythema in few patients.	Topical lactobacilli significantly improved acne and modulated microbiome composition. Findings suggest potential but require replication in larger RCTs.
Sathikulpakdee et al. / 2022^7^	Topical Probiotics	Single-blind RCT.	104 patients with mild-to-moderate acne.	To evaluate the efficacy and safety of probiotic-derived lotion compared to 2.5% benzoyl peroxide.	Lotion with cell-free supernatant of *L. paracasei* MSMC 39-1 vs. 2.5% benzoyl peroxide, applied twice daily for 4 weeks.	Both groups showed within-group reductions in inflammatory lesions, with no statistically significant difference between treatments (p = 0.23). Probiotic lotion reduced erythema from baseline (p < 0.001).	Well tolerated; fewer side effects (erythem, itching, scaling) in probiotic group.	Probiotic-derived lotion was safe, with comparable efficacy to benzoyl peroxide. Evidence remains preliminary and requires larger trials.
Casari et al. / 2022^8^	Topical Probiotics	Open-label pilot clinical study.	29 patients with mild-to-moderate acne.	To evaluate the efficacy and safety of a probiotic and hyaluronic acid-based serum.	Nightly application of serum with *L. paracasei* LiveSkin88 + hyaluronic acid for 28 days.	Significant reduction in papules/pustules at day 14 and 28 (*p* < 0.0001); erythema, skin hydration and affected facial area also improved (*p* < 0.05)	No adverse events; high patient satisfaction.	Probiotic serum improved inflammatory lesions and hydration. Preliminary evidence, requiring replication in controlled RCTs.
Karoglan et al. / 2019^9^	LBPs	Open-label Pilot Study	14 patients with mild-to-moderate acne.	To evaluate safety and efficacy of applying non-pathogenic *C. acnes* strains.	Hydrogel formulations with specific non-pathogenic *C. acnes* strains; applied for 5 weeks.	Significant reduction in non-inflammatory lesions in both groups (p = 0.029 and 0.036). No effect on inflammatory lesions.	No severe adverse effects reported	Microbiome modulation through non-pathogenic *C. acnes* strains shows promise as a potential treatment for acne.
Knödlseder et al. / 2024^10^	LBPs	Preclinical (murine model + in vitro).	5 mice (genetically modified *C. acnes* group), 9 mice (control group).	To evaluate feasibility of engineered *C. acnes* expressing NGAL for sebum modulation.	*C. acnes engineered to express NGAL, applied to murine skin and sebocyte cultures.*	Sebum production was significantly reduced in sebocyte cultures treated with NGAL. No increase in pro-inflammatory cytokines (IL-1β, IL-6, TNF-α).	No adverse effects observed	Engineered *C. acnes* shows promise for targeted sebum modulation; evidence remains limited to in vitro and murine models.
AOBiome / published data 2022^11^	LBPs	Phase IIb RCT, double-blind, placebo-controlled.	358 adult patients with mild-to-moderate acne.	To evaluate efficacy and safety of topical *Nitrosomonas eutropha* B244.	*N. eutropha* B244 topical spray applied daily for 12 weeks.	Significantly higher proportion achieving IGA success (score 0–1) vs placebo (OR 2.45; 95% CI 1.08–5.56; p = 0.03); inflammatory lesions not significantly different.	Well-tolerated, with no treatment-emergent adverse events reported.	The topical application of *Nitrosomonas eutropha* significantly improved acne, presenting an alternative for managing mild-to-moderate acne.
Han et al. / 2022^12^	Topical Postbiotic	Randomized, placebo-controlled, split-face study.	20 patients with mild-to-moderate acne (IGA 2–3).	To evaluate the efficacy and safety of a lotion containing *Enterococcus faecalis* CBT SL-5 extract.	*E. faecalis* CBT SL-5 lotion applied to one side of the face; vehicle lotion to the other, twice daily for 4-weeks.	Significant improvement in global acne scores at all time points (week 2 *p*=0.009; week 4 *p*=0.005; week 6 *p*<0.001); reduced *C. acnes* density on treated side.	No serious adverse effects reported.	Topical postbiotics with *E. faecalis* CBT SL-5 extract improved acne and reduced *C. acnes* density. Promising results, but larger RCTs are required.
Ho et al. / 2022^13^	Topical Postbiotic	RCT	20 patients with acne vulgaris.	To evaluate the efficacy of a co-fermented postbiotic with collagen in improving acne, reducing inflammation, and promoting skin healing.	Co-fermented topical gel (TYCA06/AP-32/CP-9 with collagen), applied twice daily for 4-weeks.	Significant improvement in skin hydration (+4.5%; *p*<0.05), reduced erythema (−7.1%; *p*<0.01) and brown spots (−8.7%; *p*<0.001); nonsignificant sebum reduction (−6.2%); downregulation of TSLP and IL-33.	Well tolerated, no adverse effects reported	The postbiotic formulation was safe and showed improvements in hydration, erythema, and pigmentation. Further validation in larger trials is needed.
Guerra-Tapia et al. / 2024^14^	Biotechnological Phytocomplexes	Open-label prospective study.	43 patients with truncal mild-moderate acne.	To evaluate the effect of a lotion containing a biotechnological phytocomplex, on bacterial diversity and clinical outcomes in truncal acne.	Lotion containing plant-based phytocomplexes (*C. sinensis* and *M. citrifolia* callus lysates), niacinamide 4%, succinic acid 2%, applied twice daily for 8-weeks.	Inflammatory lesions reduced by 52.1% (p = 0.006); IGA decreased by 27.6% (p < 0.001), with 60.5% of patients achieving ≥1-point improvement; *C. acnes* relative abundance decreased. Erythema reduced by 18.3% (p = 0.007); desquamation reduced by 63.8% (p = 0.02).	The treatment was well tolerated, with only two cases of mild itching reported.	Preliminary evidence of efficacy on inflammatory lesions and microbiota, but open-label design limit interpretation; RCTs are needed.
De Lucas et al. / 2024^15^	Biotechnological Phytocomplexes	Open-label, prospective study.	44 patients with mild-to-moderate acne.	To evaluate the effect of a facial cream gel containing a biotechnological phytocomplex on skin microbiota balance and clinical acne severity.	Topical application of a facial cream gel containing *C. sinensis* and *M. citrifolia* callus lysates, niacinamide 4%, succinic acid 2%, applied daily for 8-weeks.	Inflammatory lesions reduced by 47.3% (p < 0.001); non-inflammatory lesions reduced by 31.1% (p = 0.05); significant IGA improvement (p < 0.001). No 95% CI reported.	Well, tolerated with no severe adverse effects.	Phytocomplex improved both inflammatory and non-inflammatory lesions, but small open-label design and lack of CI limit conclusions; RCTs required.
Kim et al. / 2019^16^	Bacteriophage therapy	Experimental preclinical, murine model	Nine Hairless 1 (HR-1) mice.	To evaluate the effect of bacteriophage therapy on *C. acnes*-induced inflammation in a mouse acne model.	HR-1 mice were injected intradermally with *C. acnes* (10⁹ CFU/μL) followed by bacteriophage therapy in the treatment group.	Reduced inflammatory nodule size, decreased epidermal thickness and microcomedones; trends to lower CD8+ T-cells, neutrophils, IL-1β and MMP-3 (not statistically significant, no 95% CI reported).	No adverse effects observed	Early preclinical evidence that phages may reduce *C. acnes*-induced inflammation, but findings limited by lack of significant immunohistochemical effects.
Lam et al. / 2021^17^	Bacteriophage therapy	Experimental preclinical, murine model	24 male BALB/c mice with MDR *C. acnes* infection.	To evaluate the therapeutic effect of a newly isolated lytic bacteriophage (TCUCAP1) against *C. acnes*.	Intraperitoneal injection of *C. acnes*, followed by application of TCUCAP1 bacteriophage in a hydroxyethyl cellulose cream.	Significant reduction in inflammatory lesions; reduced IL-1β (p < 0.05) and caspase-3 (p < 0.01). No 95% CI reported.	No severe adverse effects reported	TCUCAP1 effectively reduced MDR *C. acnes*-induced inflammation, suggesting potential as an alternative for antibiotic-resistant acne.
Golembo et al. / 2022^18^	Bacteriophage therapy	Phase 1 randomized, double-blind, vehicle-controlled clinical trial.	75 patients with mild-to-moderate acne.	To evaluate the safety, tolerability, and ability of a topical bacteriophage gel (BX001) to reduce *C. acnes* burden on facial skin.	BX001 (three *C. acnes*-specific bacteriophages) formulated into hydroxyethyl cellulose gel, applied once daily for 4-weeks.	High-dose BX001 significantly reduced *C. acnes* load vs. vehicle (from day 14–35, p = 0.036). No lesion counts, IGA, or relapse outcomes reported; no 95% CI provided.	Well tolerated; no serious adverse events; preserved microbiome diversity.	BX001 showed targeted antibacterial effect, but clinical efficacy remains unproven; RCTs with standardized clinical endpoints are needed.
Eguren et al. / 2024^19^	Oral Probiotics	Randomized, double-blind, placebo-controlled clinical trial.	81 patients with mild-to-moderate acne (42 probiotic, 39 placebo).	To evaluate the efficacy and safety of an oral probiotic formulation in improving acne severity.	Daily capsule with *Lacticaseibacillus rhamnosus* (CECT 30031) + *Arthrospira platensis* (BEA_IDA_0074B), 1 × 10⁹ CFU/day for 12 weeks.	The probiotic group exhibited a significant reducion in non-inflammatory lesions (-8.06; 95% CI - 15.37 to -0.74; p = 0.03) and improvement in acne severity.	Well tolerated, only mild digestive discomfort reported.	Oral probiotic treatment was safe and effective, significantly improving acne severity and lesion reduction.
Rinaldi et al. / 2022^20^	Oral probiotics	Randomized, double-blind, placebo-controlled clinical trial	114 patients with mild to moderate acne.	To assess efficacy and safety of a multi-strain probiotic + botanicals on acne and microbiome balance.	Dietary supplement containing probiotics *(Bifidobacterium breve* BR03, *Lacticaseibacillus casei* LC03, *Ligilactobacillus salivarius* LS03) and botanical extracts (*lupeol from Solanum melongena* and *Echinacea* extract).	Reduction in inflammatory lesions, along with improved erythema, desquamation, and sebum secretion in the supplement group compared to placebo. *C. acnes* and *S. aureus* levels decreased, while beneficial *S. epidermidis* increased on the skin.	Well-tolerated treatment, no serious adverse events reported	The combination of probiotics and botanical extracts reduced acne severity and improved skin microbiome, supporting its potential as an adjuvant therapy for inflammatory acne.
Da Rocha et al. / 2023^21^	Oral probiotics	Randomized, double-blind, placebo-controlled clinical trial	212 patients with mild-to-moderate acne (107 treatment, 105 placebo).	To assess the efficacy of an oral probiotic combined with fixed-dose topical treatment (benzoyl peroxide + adapalene) versus the same topical treatment with a placebo.	Oral probiotic (*Lactobacillus acidophilus, Bifidobacterium lactis*) + topical adapalene 0.1% + benzoyl peroxide 2.5% for 90-days, followed by 90 days of probiotic or placebo alone.	Higher proportion achieved IGA 0–1 in probiotic group vs topical alone (p < 0.05). Significant improvement in global acne severity. 95% CIs not reported	Well tolerated, mild gastrointestinal events in probiotic group.	Oral probiotics combined with topical therapy improved outcomes and should be considered as adjuvant for acne management
Huang et al. / 2024^22^	Omega-3 Fatty Acids	Randomized controlled clinical trial + animal model study.	40 patients with moderate-to-severe acne + 20 healthy controls.	To evaluate the role of ω-3 fatty acids in acne treatment and gut microbiota modulation.	Isotretinoin alone vs. isotretinoin + ω-3 fatty acids (2400 mg/day) for 12-weeks.	GAGS scores improved (15.1 ± 4.5 vs. 18.7 ± 4.9; MD = -3.6; p = 0.02).	Well tolerated; mild gastrointestinal discomfort in some patients.	Omega-3 fatty acids may serve as an effective adjuvant to isotretinoin, potentially modulating gut microbiota and reducing inflammation.
Jung et al. / 2014^23^	Omega-3 Fatty Acids	Randomized, double-blind, controlled trial	45 patients with mild to moderate acne.	To evaluate the effects of ω-3 and GLA supplementation on acne lesion counts and severity.	Daily supplementation with ω-3 fatty acids (2000 mg/day EPA + DHA) or γ-linoleic acid (GLA, 400 mg/day) vs. control for 10-weeks.	ω-3 group: inflammatory lesions reduced 43% (from 10.1 ± 3.2 to 5.8 ± 3.4), non-inflammatory lesions reduced 20% (from 23.5 ± 9.2 to 18.9 ± 8.3). Cunliffe grade improved from 2.4 to 1.7. GLA group showed similar improvements. 95% CI not reported.	Well tolerated; some mild gastrointestinal discomfort.	Omega-3 and GLA supplementation appear safe and effective as adjuvant therapies for mild-to-moderate acne, though evidence is limited to small early-phase trials.
Yang et al. / 2021^24^	ALA-PDT	Prospective clinical study	15 patients with moderate-to-severe acne vulgaris.	To examine the effects of ALA-PDT on the skin microbiome, (impact on *C. acnes* and microbial diversity).	Four sessions of 5% ALA-PDT at two-week intervals. Skin microbiome samples were taken before treatment and before the final session.	Reduction in *C. acnes* abundance; increase in *Bacillus* and *Lactococcus*; improvement in overall microbial diversity. Shift toward a eubiotic profile. No 95% CI reported.	Well tolerated, only mild erythema observed	ALA-PDT modulated the skin microbiome by reducing *C. acnes* abundance and enhancing overall microbial diversity, supporting its role in microbiome modulation.
Guo et al. / 2023^25^	ALA-PDT	Prospective observational study	18 patients with severe acne + 8 healthy controls.	To evaluate the effects of ALA-PDT on microbiome composition.	ALA-PDT once a week for 3-weeks. Skin microbiome was analyzed before and after treatment using 16S ribosomal RNA sequencing.	Increase in microbial diversity; restoration of *Pseudomonas*, *Gordonia*, *Leptotrichia*, and *Mycobacterium* (genera often depleted in acne). *C. acnes* levels remained unchanged. No 95% CI reported.	No major adverse effects reported.	ALA-PDT modulates skin microbiota composition in severe acne patients, potentially contributing to its therapeutic effect.
Zhang et al. / 2023^26^	M-PDT	Multicenter RCT	152 patients with moderate-to-severe acne (77 M-PDT vs. 75 ISO)	To compare the efficacy and onset of action of M-PDT versus low-dose ISO in patients with moderate-to-severe acne.	Up to 5-weekly sessions of M-PDT following manual comedone extraction vs. low-dose isotretinoin (0.5 mg/kg/day) for 6-months.	M-PDT achieved 50% clinical improvement after 1-week, compared with 8-weeks for isotretinoin. Comparable efficacy at 2, 4, and 6-months. No 95% CI reported.	M-PDT caused only local skin irritation, while ISO was associated with systemic side effects in 70.6% of patients.	M-PDT offers faster onset, fewer systemic adverse effects, and comparable efficacy, making it a promising alternative.
Kim et al. / 2023^27^	SJW- PDT vs. IAA- PDT	Randomized, double-blind, split-face, vehicle-controlled clinical trial	31 patients with mild-to-moderate acne	To compare the efficacy of SJW-PDT vs. IAA-PDT for acne treatment and assess their skin rejuvenation effects.	Split-face design: one side treated with SJW-PDT and the other with IAA-PDT.	SJW-PDT reduced acne lesions by 56.5% at 1-week and 65.9% at 4-weeks; sebum production decreased by 27.9%. IAA-PDT was also effective but less pronounced. No 95% CI reported.	No reported adverse effects in both groups.	Both protocols were effective; SJW-PDT provided stronger clinical and sebosuppressive effects.
Zheng et al. / 2024^28^	Curcumin- PDT.	Experimental in vitro study.	Tested on *C. acnes* bacterial cultures and biofilms.	To assess the effect of curcumin-based PDT on *C. acnes* biofilms.	25 clinical *C. acnes* strains were tested for antibiotic resistance and biofilm formation capabilities. Biofilms were treated with curcumin-PDT.	Curcumin-PDT significantly reduced the survival of antibiotic-resistant *C. acnes.* Biofilm structure was disrupted, with increased permeability and bacterial cell death. No 95% CI reported.	Not assessed in vivo	Curcumin-based PDT showed potent anti-biofilm and antibacterial activity against *C. acnes* in vitro, supporting its potential as a future photosensitizer; clinical efficacy remains untested.

*C. Acnes: Cutibacterium Acnes*; RCT, Randomized Controlled Trial; LBPs, Live Biotherapeutic Products; *S. Aureus*: S*taphylococcus Aureus*; *S. Epidermidis: Staphylococcus Epidermidis*; IGA, Investigator Global Assessment; GAGS: Global Acne Grading System; PDT, Photodynamic Therapy; MDR, Multi-Drug-Resistant; IAA, Indole-3-Acetic Acid; ALA, 5-Aminolevulinic Acid; SJW, St. John’s Wort; ISO, Isotretinoin; M-PDT, Modified red light 5-aminolevulinic acid PDT; GLA, Gamma-Linolenic Acid.

### Pathogenesis of acne vulgaris

Acne vulgaris is a chronic inflammatory skin condition affecting the pilosebaceous units, driven by multiple factors such as hyperseborrhea, follicular hyperkeratinization, localized immune-mediated inflammation, and dysbiosis of the skin microbiome, particularly involving *Cutibacterium acnes* (*C. acnes*).^4^ The critical role of skin microbiome dysbiosis in the onset and progression of acne has reshaped therapeutic strategies. As our understanding of the interplay between the skin microbiome and host immunity deepens, the focus is shifting from traditional antibiotic treatments to innovative microbiome-targeted therapies.^29^

### Skin microbiome and acne development

The skin microbiome is a dynamic ecosystem of microorganisms, including bacteria, viruses, fungi, and their surrounding environment, that plays an essential role in maintaining skin health.^29^ Among these microorganisms, *C. acnes*, a Gram-positive anaerobic bacterium, predominates in sebaceous gland-rich areas.^30^ Typically a commensal organism, *C. acnes* contributes to skin homeostasis by lowering pH through the release of free fatty acids and by inhibiting the growth of pathogens such as *Staphylococcus aureus* and *Streptococcus* spp.^31^ However, under conditions of dysbiosis, *C. acnes* may adopt a pathogenic role, promoting skin inflammation through lipase-mediated sebum hydrolysis and free fatty acid release.^4^

The IA1 phylotype of *C. acnes* has been identified as particularly virulent due to its ability to form biofilms and activate inflammatory pathways via Th17-mediated immune responses, including Interleukin-17 (IL-17), a key cytokine in acne pathogenesis.^30^, ^32^ Additionally, *Staphylococcus* spp. have emerged as significant contributors to acne pathogenesis, acting as pathobionts or disease modulators.^33^ Dysbiosis is further exacerbated by increased seborrhea, perpetuating a cycle of chronic inflammation and immune dysregulation.^3^

### Impact of conventional acne treatments on the microbiome

**Figure 1:**
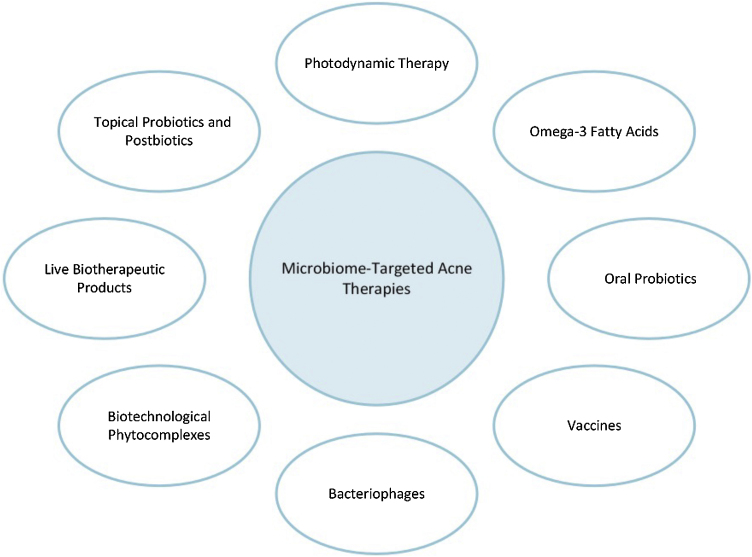
Different microbiome-targeted acne treatments.

Conventional acne treatments, such as topical retinoids, benzoyl peroxide, and oral antibiotics, primarily target *C. acnes* and modulate its associated inflammatory pathways. However, these approaches often disrupt the skin microbiome, leading to microbial imbalances that favor opportunistic pathogens and undermine cutaneous homeostasis.^34^, ^35^ The overuse of antibiotics has been linked to adverse effects such as resistance development and further microbial disruption.^34^ This increasing awareness has shifted treatment strategies from solely eradicating *C. acnes* to restoring microbiome balance and diversity, highlighting the need for innovative therapeutic alternatives.^4^, ^30^ Consequently, microbiome-focused therapies, summarized in Fig. 1, have emerged as promising strategies. These approaches aim to address dysbiosis and modulate the skin microbiome without the negative consequences often associated with traditional treatments.^36^ They emphasize restoring microbial equilibrium and reducing inflammation, representing a significant shift toward sustainable and targeted acne management.

### Non-pharmacological microbiome-targeted approaches in acne treatment

#### Topical probiotics

Topical probiotics and postbiotics have emerged as promising alternatives to antibiotics by modulating the skin microbiota.^4^ In a double-blind, placebo-controlled trial conducted in 79 patients wild mild-to-moderate acne vulgaris, Lebeer et al. (2022) evaluated a topical cream containing *Lactobacillus rhamnosus* GG, *Lactiplantibacillus plantarum* WCFS1, and *Lacticaseibacillus pentosus* KCA1 applied twice daily for eight weeks. The treatment significantly reduced *C. acnes* and *Staphylococcus* spp. colonization, while increasing *Lactobacillus* spp. abundance, likely due to decreased lipase activity and lactic acid production. Clinically, inflammatory lesion counts decreased significantly compared with placebo at weeks 4, 8, and 12, with a 34.4% reduction at week 4 in the active group versus 1.7% in the placebo group (p < 0.001). Although the exact Mean Difference (MD) was not reported, the reduction was statistically significant. Furthermore, the topical probiotic cream was well tolerated and improved skin hydration.^6^

Sathikulpakdee et al. (2022) conducted a single-blind RCT including104 patients to compare a probiotic-derived lotion containing the cell-free supernatant of *L. paracasei* MSMC 39-1 with 2.5% benzoyl peroxide. After four weeks, both groups showed significant within-group reductions in inflammatory lesion counts (p < 0.001 for both), with no statistically significant difference between treatments (p = 0.23). The probiotic lotion also significantly reduced erythema index from baseline (from 22.9 ± 1.9 to 21.4 ± 2.1 at week 4, p < 0.001), and showed a favorable tolerability profile, with fewer adverse events (7.69%) compared with benzoyl peroxide (26.92%).^7^

In a smaller open-label pilot study, Casari et al. (2022) evaluated a serum containing *Lactobacillus. paracasei* LiveSkin88 combined with hyaluronic acid in 29 patients with mild-to-moderate acne, applied nightly for 28 days. The treatment led to a statistically significant reduction in the number of papules and/or pustules (p < 0.0001). By day 14, the mean number of papules and/or pustules decreased from 7.9 ± 4.0 (mean value ± SD) at baseline to 4.7 ± 3.0 (95% CI), with improvements maintained through day 28 (mean 4.5 ± 3.8). Erythema also showed a a modest but significant improvement (p < 0.0001), while skin hydration increased from 53.5 ± 22.0 to 64.7 ± 19.9 arbitrary units (p < 0.0001; 95% CI). The facial area affected by acne significantly improved after 28 days (p < 0.05). No adverse events were reported, and patient satisfaction was high.^8^

Taken together, these trials underscore the strain-specific benefits of topical probiotics in acne management, ranging from reductions in inflammatory lesions to improvements in erythema and hydration. However, current evidence remains preliminary. Limitations include small sample sizes, short follow-up periods, heterogeneous formulations, and the fact that only one trial to date has been double-blind and placebo-controlled. Replication in larger, high-quality RCTs with standardized clinical outcomes, including lesion counts, Investigator’s Global Assessment (IGA) scores, and relapse or recurrence rates, is essential to establish the efficacy, safety, and generalizability of topical probiotics in acne care.

#### Live biotherapeutic products

Emerging literature also highlights the potential benefit of Live Biotherapeutic Products (LBPs), a novel class of therapeutics incorporating live bacteria into topical formulations designed to modulate the skin microbiota and address localized dysbiosis.^37^ Various LBP formulations using *C. acnes* strains have been investigated.

Karoglan et al. (2019) conducted an open-label pilot study in 14 patients with mild-to-moderate acne to assess whether non-pathogenic *C. acnes* strains could modulate the skin microbiome. Participants received one of two hydrogel formulations for five weeks. Non-inflammatory lesion counts decreased significantly in both groups: from 62 to 37 (mean reduction – 25 lesions; p = 0.029) and from 54 to 35 (mean reduction − 19 lesions; p = 0.036). Inflammatory lesion counts showed no significant changes (p > 0.26). Microbiome analysis revealed colonization by the applied strains, with no irritation or flare-ups reported, supporting the safety and feasibility of this microbiome-modulating approach.^9^

Knödlseder et al. (2024) developed an engineered *C. acnes* strain expressing Neutrophil Gelatinase-Associated Lipocalin (NGAL), a protein involved in lipid regulation. In sebocyte cultures, NGAL reduced sebum production by approximately twofold within 48 hours, an effect comparable to isotretinoin 10 μM (p < 0.0001). NGAL produced by recombinant *E. coli* reduced sebum 1.7-fold but was less effective than the engineered *C. acnes*. Importantly, no cytotoxicity or apoptosis was observed. In murine models, the engineered strain colonized the skin and maintained NGAL expression without increasing pro-inflammatory cytokines such as IL-1β, IL-6, or TNF-α, underscoring its potential as a safe approach for targeted sebum modulation.^10^

AOBiome’s Phase IIb trial (NCT02832063) investigated the topical application of *Nitrosomonas eutropha* B244, an ammonia-oxidizing bacterium, in 358 patients with mild-to-moderate acne. The randomized, double-blind, placebo-controlled design tested treatment for 12-weeks. Compared to placebo, B244 yielded a significantly higher proportion of participants reaching Investigator’s Global Assessment (IGA) success, defined as a post-baseline score of 0 or 1 (Odds Ratio = 2.45; 95% CI 1.08–5.56; p = 0.03). Inflammatory lesion counts showed a numerical, but not statistically significant, improvement versus placebo. The formulation was well tolerated, with no treatment-emergent adverse events reported.^11^

These findings underscore the potential of LBPs to introduce a paradigm shift in acne therapy, focusing on microbial composition and host-microbe interactions. However, current evidence remains preliminary. Karoglan et al. provided only pilot open-label data without a control group (n = 14). Knödlseder et al. demonstrated promising effects, but only in vitro and in murine models. AOBiome’s Phase IIb trial represents the most robust clinical evidence to date, though its primary outcome was not achieved in the intention-to-treat analysis. Larger, standardized, and controlled trials are warranted to confirm the clinical efficacy, durability, and safety of LBPs in acne management.

#### Topical postbiotics

Postbiotics, defined as preparations of inanimate microorganisms and/or their components that provide health benefits to the host, have demonstrated multiple benefits for the skin microbiome, including enhancing skin barrier function and promoting the growth of beneficial endogenous bacteria.^38^ For acne management, various postbiotics have shown potential in restoring microbial balance and reducing acne severity through their anti-inflammatory and antimicrobial effects.^39^

Emerging evidence highlights the effectiveness of fermentation-derived products, such as *Enterococcus faecalis* CBT SL-5 extract, as a safe and well-tolerated topical option for reducing acne severity and addressing skin microbiome dysbiosis.^12^ Han et al. (2022) conducted a randomized, placebo-controlled, split-face comparative study in 20 patients with mild-to-moderate acne. Participants applied a lotion containing *E. faecalis* CBT SL-5 extract twice daily for four weeks. The treated side showed significantly greater IGA improvement scores compared with the control side at all time points: week 2 (1.65 ± 0.81 vs 1.05 ± 0.51; p = 0.009), week 4 (1.80 ± 0.83 vs 1.15 ± 0.49; p = 0.005), and week 6 (2.15 ± 0.88 vs 1.05 ± 0.51; p < 0.001). Additionally, *C. acnes* density decreased significantly on the treated side, whereas no significant changes occurred on the control side. No treatment-related adverse effects were reported. Confidence intervals were not reported in the original publication.^12^

Similarly, Ho et al. (2022) performed a randomized clinical trial evaluating a co-fermented postbiotic formulation containing probiotic strains TYCA06, AP-32, and CP-9 combined with collagen in 20 patients with acne vulgaris. The formulation was applied twice daily for four weeks. The formulation significantly improved skin hydration (+ 4.5% from baseline; p < 0.05), reduced erythema intensity by 7.1% (*p* < 0.01), and decreased the number of brown spots by 8.7% (*p* < 0.001). Sebum production showed a nonsignificant reduction of 6.2%. In vitro assays demonstrated downregulation of Thymic Stromal Lymphopoietin (TSLP) and Interleukin-33 (IL-33) expression in human keratinocyte cultures, supporting an anti-inflammatory effect. The treatment was well-tolerated with no adverse events. Confidence intervals were not reported.^13^

Although these early-phase trials suggest potential benefits, clinical evidence supporting topical postbiotics in acne remains exploratory. Both Han et al. and Ho et al. relied on small samples and short follow-up durations, lacked standardized acne endpoints, and assessed heterogeneous formulations. The split-face design used by Han introduces observer bias, while Ho et al. primarily evaluated surrogate and cosmetic outcomes. To establish the translational applicability of topical postbiotics, future studies should employ adequately powered randomized controlled trials with standardized clinical and microbiome-informed outcomes.

#### Biotechnological phytocomplexes

Recent studies have evaluated biotechnological phytocomplexes in acne treatment, highlighting their antibiofilm and microbiota-modulating properties. These formulations combine metabolomes derived from plant stem cell cultures of *Camellia sinensis* and *Morinda citrifolia*. *C. sinensis* exhibits anti-inflammatory effects by reducing IL-6, IL-8, TNF-α, and CXCL levels, along with antiseborrheic and antibiofilm properties. Meanwhile, *M. citrifolia* inhibits biofilm formation and blocks bacterial communication, contributing to microbiota rebalancing.^14^, ^15^

A novel phytocomplex lotion containing Canonia allysis©, niacinamide 4.00% and succinic acid 2.00% was evaluated in two open-label trials in patients with mild-to-moderate facial and truncal acne.^14^, ^15^ Guerra-Tapia et al. (2024) conducted a prospective open-label study in 43 subjects with mild-to-moderate truncal acne, treated for eight consecutive weeks (56 days). The application of this phytocomplex reduced inflammatory lesions by 52.1% (p = 0.006) and acne severity by 27.6% on the IGA scale, with 60.5% of patients achieving ≥ 1-point improvement (p < 0.001). Microbiome analysis showed a significant decrease in *C. acnes* relative abundance (66.4% to 58.1%, p = 0.009), indicating partial microbial rebalancing. Erythema decreased by 18.3% (p = 0.007) and desquamation by 63.8% (p = 0.02). No serious adverse events were reported, supporting favorable tolerability.^14^

Similarly, De Lucas et al. (2024) conducted an open-label study in 44 patients with mild-to-moderate facial acne, treated for eight weeks. Inflammatory lesions decreased by 47.3% (p < 0.001), while non-inflammatory lesions decreased by 31.1%, with borderline significance (p = 0.05). IGA scores also improved significantly at day 56 (p < 0.001). No serious adverse events occurred.^15^

Despite these encouraging findings, the clinical evidence supporting biotechnological phytocomplexes remains preliminary. Both studies were open-label, non-randomized, with modest sample sizes (n = 43 and n = 44), lacked control groups, and did not report 95% Confidence Intervals, limiting interpretation of effect sizes. Moreover, both evaluated the same proprietary formulation and were industry-sponsored, raising concerns regarding potential conflicts of interest and publication bias. Although reported outcomes include statistically significant reductions in acne severity and microbial imbalance, the lack of independent, adequately powered randomized controlled trials with standardized clinical and microbiome-informed endpoints limits external validity and underscores the need for further research to establish their therapeutic role in acne care.

#### Bacteriophages

Controlling the inflammatory response is a primary goal in acne treatment, particularly given the rise of antimicrobial resistance. Bacteriophage therapy has emerged as a potential alternative due to its ability to specifically target *C. acnes* while preserving beneficial microbes. This strategy may help restore microbial balance and maintain skin microbiota homeostasis.^40^, ^41^

Preclinical studies provide the initial evidence for this approach. Kim et al. (2019) tested *C. acnes*-specific phages in a murine acne model, showing a reduction in inflammatory nodule size compared to controls. Histological analyses revealed decreased epidermal thickness and fewer microcomedone-like cysts, while immunohistochemical results suggested reduced CD8+ T-cell and neutrophil infiltration, as well as lower IL-1β and MMP-3 expression. However, most of these changes were not statistically significant, and no 95% Confidence Intervals were reported, limiting the interpretation of effect sizes.^16^ Similarly, Lam et al. (2021) evaluated the therapeutic phage TCUCAP1 in mice, observing significant reductions in nodule size and epidermal hyperplasia, together with decreased IL-1β (p < 0.05) and caspase-3 (p < 0.01). Only p-values were reported without confidence intervals.^17^

The only human evidence to date comes from Golembo et al. (2022), a randomized, double-blind, vehicle-controlled Phase 1 trial testing a topical gel containing the bacteriophage cocktail BX001 in patients with mild-to-moderate acne. The high-dose formulation significantly reduced *C. acnes* load compared with baseline and vehicle (p = 0.036), with effects observed from day 14 to day 35. The low-dose formulation did not show significant effects. Importantly, the study did not report lesion counts, Investigator’s Global Assessment (IGA) scores, or relapse outcomes, and no 95% Confidence Intervals were provided. BX001 was well tolerated, with no serious adverse events, and did not alter overall microbiome diversity.^18^

Bacteriophage therapy for acne remains early-phase, with encouraging but limited preclinical and Phase 1 findings. Current evidence is restricted to microbiological and histological outcomes, without robust clinical endpoints. To establish their therapeutic potential, durability of effect, and relevance for acne management, independently conducted randomized controlled trials with standardized clinical and microbiome-informed outcomes, including lesion counts, IGA scores, and relapse or recurrence rates, are required.

#### Vaccines

Vaccines represent another innovative strategy targeting acne pathogenesis. By focusing on virulence factors such as the CAMP factor and sialidase, these approaches aim to neutralize *C. acnes* pathogenicity and reduce the inflammatory response while preserving commensal microbiota and minimizing side effects.^41^, ^42^, ^43^ Sanofi is currently conducting a Phase I/II randomized, double-blind, placebo-controlled clinical trial (NCT06316297) evaluating the safety, immunogenicity, and efficacy of an mRNA acne vaccine targeting *C. acnes* virulence factors. This ongoing trial includes healthy participants aged 18–45 years with moderate-to-severe acne and is assessing different dose levels administered intramuscularly.^44^

Additionally, a completed Phase I trial (NCT05131373) investigated the safety, tolerability, and immunogenicity of ORI-A-ce001, an acne vaccine for treating moderate facial acne vulgaris in adults. This multicenter, randomized, double-blind, placebo-controlled trial evaluated various dose levels in adults aged 18-years and older. In addition to assessing safety and immune responses, the study collected preliminary data on the vaccine’s efficacy, including its impact on inflammatory and non-inflammatory lesions, acne severity, and skin microbiome composition.^45^ These findings provide valuable insights into the potential role of vaccines as a novel therapeutic approach for acne management.

Despite their conceptual appeal, acne vaccines remain in the early stages of clinical development. Both referenced trials are Phase I studies primarily focused on safety and immunogenicity, with no peer-reviewed efficacy results available to date. Larger, well-designed clinical trials are necessary to demonstrate meaningful improvements in acne severity, characterize immune durability, and assess real-world applicability.

#### Gut-skin-axis interventions

The gut-skin axis highlights the bidirectional relationship between the gastrointestinal system and skin health, mediated by immune regulation, the microbiota, and neuroendocrine pathways. The gut microbiota plays a pivotal role in acne development and overall skin health by modulating immune responses and systemic inflammation. Disruptions such as dysbiosis or impaired intestinal barrier function can exacerbate skin conditions, including acne. Although the contribution of the gut microbiota to acne pathogenesis remains underexplored, targeting gut health through integrative approaches holds promising potential for improving skin outcomes.^46^

a) Oral probiotics:

Eguren et al. (2024) conducted a 12-week randomized, double-blind, placebo-controlled clinical trial evaluating an oral probiotic supplement containing *Lacticaseibacillus rhamnosus* (CECT 30031) and the the cyanobacterium *Arthrospira platensis* (BEA_IDA_0074B) at 1 × 10^9^ CFU per daily dose in 81 individuals with mild-to-moderate acne vulgaris. Non-inflammatory lesions decreased significantly versus placebo group, with a difference of −8.06 lesions (95% CI −15.37 to −0.74; p = 0.03). Improvements were also observed in acne severity, with 50% of probiotic-treated patients improving by at least one Acne Global Severity Scale (AGSS) category, and 42.5% achieving ≥ 30% reduction in Global Acne Grading System (GAGS) scores (both p < 0,05). Inflammatory and total lesion counts showed no significant differences between groups (p = 0.040). The treatment was well tolerated, with only mild digestive events reported.^19^

Rinaldi et al. (2022) conducted an 8-week randomized, double-blind, placebo-controlled trial evaluating a dietary supplement containing *Bifidobacterium breve* BR03 (DSM 16604), *Lacticaseibacillus casei* LC03, and *Ligilactobacillus salivarius* LS03 combined with botanical extracts (lupeol and *Echinacea*) in patients with mild-to-moderate acne. The intervention significantly reduced inflammatory lesions (-56.7% vs. -18.9% in placebo; p < 0.05), decreased sebum secretion and desquamation, and favorably modulated the skin microbiota by reducing *C. acnes* and *S. aureus* while increasing *S. epidermidis*. However, 95% Confidence Intervals were not reported, limiting the interpretation of the magnitude of these effects.^20^

Da Rocha et al. (2023) conducted a 180-day randomized, double-blind, placebo-controlled trial in 212 patients with mild-to-moderate acne, evaluating an oral probiotic supplement (*Lactobacillus acidophilus* and *Bifidobacterium lactis*) combined with a fixed-dose topical regimen of adapalene 0.1% and benzoyl peroxide 2.5%. The combined approach significantly improved outcomes, with a higher proportion of patients achieving an IGA score of 0 or 1 compared with topical therapy alone (p < 0.05). The probiotic regimen was well tolerated, but as with the Rinaldi trial, no 95% Confidence Intervals were provided.^21^

Although the clinical evaluation of oral probiotics in acne has progressed, substantial variability in strain composition, outcome measures, and treatment duration limits external validity and reproducibility. Mechanistic endpoints and microbiome-informed outcomes were inconsistently assessed across studies. Despite these limitations, the consistent safety profile and randomized, placebo-controlled designs underscore their potential. Standardized protocols incorporating both clinical and microbiome endpoints, with clear reporting of effect sizes and 95% Confidence Intervals, are necessary to define the therapeutic role of oral probiotics in acne care.

b) Omega-3 fatty acids:

The fatty acids ω-3 and ω-6 are thought to have potential utility in acne through anti-inflammatory effects.^47^ Huang et al. (2024) conducted a 12-week randomized trial in 40 patients with moderate-to-severe acne treated with isotretinoin with or without ω-3 fatty acids (2400 mg/day). The combination group achieved greater reductions in Global Acne Grading System (GAGS) scores (mean 15.1 ± 4.5) compared with isotretinoin alone (18.7 ± 4.9), with a mean difference of -3.6 points (p = 0.02).^22^ These findings align with the results previously reported by Jung et al. (2014), who evaluated 45 patients randomized to ω-3 fatty acids (2000 mg/day EPA + DHA), γ-linoleic acid (GLA, 400 mg/day), or control for 10 weeks. The ω-3 group showed a 43% reduction in inflammatory lesions (10.1 ± 3.2 to 5.8 ± 3.4) and a 20% reduction in non-inflammatory lesions (23.5 ± 9.2 to 18.9 ± 8.3), both p < 0.05. Similar improvements were observed in the GLA group, while no significant changes occurred in controls. Acne severity (Cunliffe grade) improved from 2.4 to 1.7 in the ω-3 group and from 2.3 to 1.8 in the GLA group (p < 0.05).^23^

Collectively, these findings suggest that fatty acid supplementation may reduce acne lesion counts and severity; however, the lack of reported confidence intervals, small sample sizes, and short treatment durations limit interpretation of effect sizes and generalizability. In Huang et al., the concurrent use of isotretinoin precludes definitive attribution of effects to ω-3 fatty acids, and microbiome modulation was inferred rather than directly assessed. Similarly, Jung et al. lacked detailed reporting on blinding procedures and did not evaluate gut microbiota endpoints. Overall, although the anti-inflammatory role of fatty acids in acne pathophysiology remains biologically plausible, larger, independently replicated trials with standardized endpoints and reporting of 95% CIs are required to confirm their clinical efficacy and clarify underlying mechanisms.

#### Photodynamic therapy

Photodynamic Therapy (PDT) is a non-invasive, targeted modality for inflammatory acne. It involves the application of a topical photosensitizer, most commonly Aminolevulinic Acid (ALA), followed by irradiation with a specific light source.^48^ The most frequently used wavelength is red light (630 nm), which penetrates deeper into the skin and activates Protoporphyrin IX (PpIX), the metabolite of ALA. Upon activation, PpIX generates Reactive Oxygen Species (ROS), inducing selective cytotoxicity in sebaceous glands and reducing sebum production.^49^ Beyond sebosuppression, ALA-PDT has demonstrated anti-inflammatory and microbiome-modulating effects, including disruption of *Cutibacterium acnes* biofilms and restoration of microbial diversity.^50^, ^51^ PDT has also been shown to downregulate Toll-Like Receptors (TLR-2 and TLR-4) and decrease pro-inflammatory cytokine production in sebaceous glands and the epidermis.^52^

a) ALA-PDT and microbiome modulation:

Yang et al. (2021) conducted a prospective study evaluating microbiome changes in acne patients before and after ALA-PDT. Treatment reduced *C. acnes* abundance (p = 0.037), increased *Bacillus* (p = 0.012) and *Lactococcus* (p = 0.022), and enhanced overall microbial diversity (p = 0.003), suggesting a shift toward an eubiotic environment resembling healthy skin.^24^

Guo et al. (2023) investigated ALA-PDT utilizing a 630 ± 5 nm LED light source in patients with severe acne. This intervention caused a gradual increase in the relative abundance of genera such as *Pseudomonas*, *Gordonia*, *Leptotrichia*, and *Mycobacterium*, microbes that were found at lower levels in severe acne patients compared to healthy individuals. Notably, the study found no statistically significant difference in the relative abundance of *C. acnes* among the healthy control, pretreatment, and posttreatment groups. These findings suggest that PDT’s effects extend beyond bacterial suppression to include microbiome restoration.^25^ Neither study provided 95% Confidence Intervals, limiting the interpretation of effect sizes.

b) Modified PDT protocols compared to standard therapy:

Zhang et al. (2023) performed a randomized multicenter trial comparing modified ALA-PDT (M-PDT) with low-dose isotretinoin in patients with moderate-to-severe acne. M-PDT achieved 50% clinical improvement after just 1 week, compared with 8-weeks for isotretinoin (p < 0.001). Adverse effects were limited to local skin irritation in the M-PDT group, while 70.67% of patients receiving isotretinoin experienced systemic side effects. Confidence intervals were not reported.^26^

c) Novel Photosensitizers:

Recent studies have explored natural photosensitizers as alternatives to ALA for acne management. Kim et al. (2023) conducted a randomized split-face trial comparing *St. John’s Wort* (SJW)-PDT with Indole-3-Acetic Acid (IAA)-PDT. SJW-PDT reduced acne lesions by 56.5% at 1-week and 65.9% at 4-weeks (both p < 0.001), with sebum production decreasing by 27.9% (p = 0.012) and no adverse events reported; however, confidence intervals were not provided.^27^ Zheng et al. (2024) evaluated curcumin-based PDT in vitro against *C. acnes* biofilms. The treatment demonstrated dose-dependent suppression of bacterial viability and potent anti-biofilm activity, highlighting curcumin’s potential as a natural photosensitizer for acne vulgaris, particularly in cases involving antibiotic resistance.^28^

Although PDT shows promising antimicrobial, anti-inflammatory, and microbiome-modulating effects in acne, available studies are limited by small sample sizes, heterogeneity in photosensitizers, protocols, and endpoints, and the absence of reported confidence intervals. While early findings suggest efficacy and safety, robust multicenter randomized controlled trials with standardized clinical and microbiome-informed outcomes, including lesion counts, Investigator’s Global Assessment (IGA), and relapse rates, are required to establish PDT’s therapeutic role in acne care.

## Conclusion

Acne management is undergoing significant advancements with the emergence of innovative therapies targeting microbiome modulation through non-pharmacological approaches. This strategy offers a promising avenue to restore microbial homeostasis, mitigate inflammation, and reduce reliance on conventional pharmacologic agents. The transition from traditional treatments to microbiome-focused interventions reflects a paradigm shift in acne care. These developments highlight the growing relevance of personalized, mechanism-driven therapies designed to enhance efficacy and improve patient outcomes.

Although preliminary data are promising, the evidence base remains limited by small samples, methodological weaknesses, and reliance on preclinical models. Well-designed, multicenter randomized controlled trials with standardized clinical and microbiome-informed endpoints are needed to establish the therapeutic value, long-term safety, and translational relevance of these interventions. Advancing this next phase of research is essential to move microbiome-modulating therapies from experimental innovation to standard-of-care options in acne management.

## ORCID ID

Rodrigo Funes-Ferrada: 0009-0007-3361-5107

Fernando Valenzuela: 0000-0003-1032-9347

## Research data availability

The entire dataset supporting the results of this study was published in this article.

## Financial support

None declared.

## Authors' contributions

Valentina Burckhardt-Bravo: The study concept and design; data collection, or analysis and interpretation of data; writing of the manuscript or critical review of important intellectual content; effective participation in the research guidance; critical review of the literature; final approval of the final version of the manuscript.

Rodrigo Funes-Ferrada: Writing of the manuscript or critical review of important intellectual content; effective participation in the research guidance; critical review of the literature; final approval of the final version of the manuscript.

Fernando Valenzuela: The study concept and design; data collection, or analysis, and interpretation of data; writing of the manuscript or critical review of important intellectual content; effective participation in the research guidance; intellectual participation in the propaedeutic and/or therapeutic conduct of the studied cases; critical review of the literature; final approval of the final version of the manuscript.

## Conflicts of interest

None declared.
